# Exploring Acceptability, Barriers, and Facilitators for Digital Health in Dermatology: Qualitative Focus Groups With Dermatologists, Nurses, and Patients

**DOI:** 10.2196/57172

**Published:** 2024-09-03

**Authors:** Patrick Reinders, Matthias Augustin, Anastasia Fleyder, Marina Otten

**Affiliations:** 1 Institute for Health Services Research in Dermatology and Nursing (IVDP) University Medical Center Hamburg-Eppendorf (UKE) Hamburg Germany

**Keywords:** digitalization, digital health interventions, UTAUT, unified theory of acceptance and use of technology, barriers and facilitators, dermatology, qualitative research, focus groups, mobile phone

## Abstract

**Background:**

Although several digital health interventions (DHIs) have shown promise in the care of skin diseases their uptake in Germany has been limited. To fully understand the reasons for the low uptake, an in-depth analysis of patients’ and health care providers’ barriers and facilitators in dermatology is needed.

**Objective:**

The objective of this study was to explore and compare attitudes, acceptability, barriers, and facilitators of patients, dermatologists, and nurses toward DHIs in dermatology.

**Methods:**

We conducted 6 web-based focus groups each with patients (n=34), dermatologists (n=30), and nurses (n=30) using a semistructured interview guide with short descriptions of DHIs described in the literature. A content analysis was performed using deductive constructs, following the unified theory of acceptance and use of technology framework, and inductive categories.

**Results:**

Patients identified many positive performance expectancies, such as reduced travel times and improvement in follow-up appointments. Dermatologists also stated positive effects (eg, promotion of standardized care), but also negative implications of health care digitalization (eg, increased workload). All stakeholders reported that a DHI should bring additional value to all stakeholders. A lack of digital competence among patients was identified as the major barrier to adoption by all 3 groups. Nurses and dermatologists want apps that are easy to use and easy to implement into their daily routines. Trust in selected institutions, colleagues, and physicians was identified as a facilitator. Patients reported their dependence on the dermatologists’ acceptance. All groups expressed concerns about data privacy risks and dermatologists stated insecurities toward data privacy laws.

**Conclusions:**

To ensure successful digitalization in dermatology, apps should be user-friendly, adapted to users’ skill levels, and beneficial for all stakeholders. The incorporation of dermatologists’ perspectives is especially important as their acceptance may impact use among patients and nurses. DHIs should ensure and be transparent about data privacy. The found barriers and facilitators can be used for implementation strategies.

## Introduction

Skin diseases, such as atopic dermatitis, psoriasis, acne, skin cancer, and urticaria, are among the most frequent medical conditions in Europe [1,2]. In Germany, about 26.75% (11,291 /42,215) of adults have a dermatological condition that requires further examination by a dermatologist, causing high use of health care services [2-4]. The resulting time constraints restrict possibilities for shared decision-making and impair timely access to care [5,6]. Demographic change will put additional pressure on the system in the near future [7].

Digital health interventions (DHIs) through information and communication technologies can support the provision of care [8]. In dermatology, a wide array of DHIs is available to patients and health care providers across numerous indications, each offering various features [9]. Given the visual nature of many dermatological assessments, the integration of telemedicine and artificial intelligence can support diagnoses [10,11]. Digitally supported self-management strategies may be beneficial, as many chronic dermatological conditions such as atopic dermatitis and psoriasis exist, where lifestyle adjustments can lead to positive outcomes [12]. Disease monitoring apps could also become essential in dermatology. These apps support both patients and physicians by enabling them to track disease progression through image documentation, patient-reported outcomes, and access to digital medical records, including laboratory results [13,14]. All apps can improve communication, data availability, efficiency, patient-centered care [15], and treatment adherence in dermatology [16].

Despite the variety of DHIs in the literature, their adoption in the field of dermatology remains limited. Although a guideline and reimbursement for teledermatological services exist, only 40% of dermatologists offered these services during the height of the COVID-19 pandemic in Germany [17-19]. In other countries, the number exceeded 80% [19,20]*.* When considering actual usage, published data indicate that only 7.6% (60/792) of dermatologists frequently used real-time teledermatology, with more precise statistics currently unavailable. Other DHIs are used more commonly, such as the electronic appointment reminder (“frequently used” by 212/792, 27% of dermatologists), but still used by a minority of dermatologists [21].

A recent systematic literature analysis identified key barriers to the implementation of DHIs in general health care worldwide including limited knowledge of DHIs among physicians and patients, unclear benefits for participants, and financing issues related to reimbursement and cost coverage for patients [22]. Acceptability and attitude of stakeholders, including patients, nurses, and dermatologists, play an important role in the adoption of DHIs. Whereas patients in Germany have a general interest in digital health and a willingness to share their data with dermatologists [23,24] the acceptability of German dermatologists on electronic health records is lower [25]. Nurses’ acceptability and competencies are vital for the successful implementation of DHIs because they play a pivotal role in assisting physicians by processing patient data, coordinating, and communicating with patients, educating them on DHIs, preparing data for consultations, and seamlessly integrating DHIs into clinical workflows [26]. Yet they are frequently overlooked and inadequately addressed in the literature [26]. To gain a deeper understanding of the acceptability of the 3 groups, an in-depth analysis of patients’ and health care providers’ barriers and facilitators in dermatology is needed. The perspectives identified can then be used to develop tailored interventions and implementation strategies for DHIs [27].

The objective of this study was to explore and exploratively compare attitudes, acceptability, barriers, and facilitators of patients, dermatologists, and nurses toward DHIs in dermatology.

## Methods

### Study Design

We conducted web-based focus groups with dermatologists, dermatology nurses, and patients with skin conditions via a video conference platform Cisco Webex (Cisco Systems). Focus groups are a qualitative research method that provides a deeper understanding of shared experiences and opinions by facilitating an interaction between participants [28]. We ensured the quality of the web-based focus groups by following the STEER (Stability of Group Numbers, Technology, Environment, Evaluation, and Recruitment) guidelines [29]. The COREQ (consolidated criteria for reporting qualitative research) was followed in this study reporting when applicable (Multimedia Appendix 1) [30].

### Material

The focus groups were conducted using a semistructured interview guide based on literature research and developed among a team of health scientists and dermatologists (Multimedia Appendix 2). First, questions were asked on the current status and issues in providing or receiving dermatological care. After that, a short description of five common DHIs was given (1) treatment reminders for adherence [16], (2) self-support tools and webpages [12], (3) store-and-forward teledermatology in different settings (with known and unknown dermatologists) [31,32], (4) eHealth portals for disease monitoring via active data collection (including patient-reported outcomes) [13], and (5) live-interactive video consultations [10]

In all focus groups, participants were asked to express whether they could imagine using the presented DHI, identify additional features they deemed necessary from their perspective, and pinpoint any aspects that might deter them from using the DHI (Multimedia Appendix 2). In addition, general aspects of DHIs were investigated with questions on data security, data ownership, evidence, and their impact on the patient-physician relationship.

### Participant Selection and Recruiting

To gather a broad range of perspectives on DHIs, we purposefully selected participants: dermatologists and nurses (German: Medizinische Fachangestellte or Gesundheits- und Krankenpfleger*innen) were selected based on type of workplace (clinic or practice), location of workplace (East, West, North, or South Germany and urban or rural), age group (<39, 40-49, and +50 years), and gender. Participants were invited to participate by field managers of Novartis Pharma GmbH and were compensated for their participation. A total of 33 dermatologists and 34 nurses were willing to participate, out of which 7 did not attend a focus group and without providing a specific reason.

Patients were selected based on diagnosis: psoriasis, atopic dermatitis, skin cancer, acne, hidradenitis suppurativa, and chronic wounds. Decision on indications was made to cover a wide range of dermatological care from chronic inflammatory skin diseases (eg, psoriasis and atopic dermatitis) via chronic wounds to skin cancer. Patients were invited from two sources: (1) offline via the dermatological outpatient clinic at the University Medical Center Hamburg-Eppendorf (UKE) and rural dermatological practice in the Hamburg area and (2) web-based via a patient association (Deutscher Psoriasisbund eV) by sending out an email to members. Inclusion criteria were aged ≥18 years and proficiency in German and technical equipment to participate in a video conference. Patients received an allowance for their participation. We enlisted 41 patients willing to participate, out of which 7 patients did not participate in the focus groups. Of those, 3 cited scheduling conflicts and 4 did not state any specific reason.

The researchers had no personal acquaintance with any of the participants before the focus groups.

### Ethical Considerations

The local psychological ethics committee at the UKE (Lokale Psychologische Ethikkommission am Zentrum für Psychosoziale Medizin des University Medical Center Hamburg-Eppendorf) approved this study (LPEK-0250). Participants received study information via email and gave their informed consent in a mandatory 1:1 video call before the respective focus group. Their anonymity was ensured by a personally chosen acronym during the video conference. The study was conducted following the Declaration of Helsinki.

### Data Collection

Preceding the focus groups, we conducted a mandatory 1:1 video call to (1) introduce the researcher and explain their motivation for the research project, (2) ensure participants’ technical proficiency, (3) provide information about the research project, (4) answer any questions, (5) record oral consent as described above, (6) explain ground rules for the web-based focus groups, and (7) collect sociodemographic data.

In total, 2 researchers of the UKE (health scientists) participated in each focus group: one to moderate the session and guide the discussion; the other to ensure a smooth organizational procedure and take field notes. MO (PhD and lead of research group telemedicine and digital health at UKE) or PR (PhD student and research associate in research group telemedicine and digital health at UKE) moderated the focus groups. All focus groups were scheduled with 5 to 7 participants for 1.5 hours and were conducted between February and April 2021. The recordings had an average length of 1:28 hours (SD 0.19) with a range from 0:49 hours and 1:59 hours.

We initially planned at least 6 focus groups per stakeholder group with the option to conduct further groups until data saturation would have been reached. The data saturation was discussed among the researchers during the analysis and was defined as “the degree to which new data repeat what was expressed in previous data” [33]. After the fourth focus group, approximately 70%-80% of topics were repeated within all 3 stakeholder groups, resulting in the conduction of 6 focus groups each.

### Data Analysis

All focus groups were fully recorded via audio and transcribed for qualitative content analysis. Data analysis was conducted following the recommendations by Elo and Kyngäs [34] using content analysis and the software NVivo (version 12; Lumivero). Overarching constructs were deductively based on the unified theory of acceptance and use of technology (UTAUT) model (Textbox 1). The UTAUT model aims to explain the acceptance and usage of technology and is based on 4 major constructs: performance expectancy, effort expectancy, facilitating conditions, and social influence. Another relevant construct associated with the model is the attitude toward using technology [35]. The UTAUT was chosen, as the model is one of the most frequently applied theories to explain acceptance toward technology [36] in the health care context and has proven itself in qualitative research (Textbox 1) [37,38]. Main categories and subcategories were initially derived inductively on an individual DHI (eg, treatment reminder) or topic level (eg, evidence or data privacy). In a subsequent step, a category system was developed (constructs deductively based on UTAUT), where individual DHI and topic-specific inductive categories were abstracted to identify attitudes, acceptability, barriers, and facilitators. For this study, the main and subcategories for the individual DHIs were not further analyzed, categories were formed across all. Constructs, main categories, subcategories, and quotations were openly discussed in multiple sessions between researchers (MO, PR, and AF). Similar main categories and subcategories across the 3 stakeholder groups were aligned in wording to allow for comparison between groups. The coding was carried out by PR supported by AF and MO.

**Description of deductive constructs according to the unified theory of acceptance and use of technology model.**

**Attitudes toward technology**

- Liking or disliking digital health interventions (DHIs), seeing the need for DHI, and willingness to use DHI in the future.

**Performance expectancy**

- Expected usefulness of technology, productivity, and career prospects.

**Effort expectancy**

- Expected ease or difficulty of use from an individual perspective.

**Facilitating conditions**

- Facilitator or barrier associated with resources, knowledge, compatibility with current routines, availability of assistance, and special features of the DHI.

**Social influence**

- Opinion of important others, organizational support, trust in recommendations of colleagues, societies (eg, patient or medical societies), or patients.

## Results

### Characteristics of Participants

The number of participants per focus group ranged between 4 and 7 participants. The 30 dermatologists who participated in 1 of the 6 focus groups were between 34 and 69 (mean 51.3, SD 8.4) years, and 12/30 (40%) were women (Table 1). Dermatologists predominantly worked in an outpatient practice (23/30, 77%), a minority in both settings (6/30, 20%), or in an outpatient clinic (1/30, 3%). All 4 regions were represented with a range from 12 (40%) dermatologists from western German states to 5/30 (17%) dermatologists from eastern German states. The 30 nurses were between 23 and 60 (mean 37.5, SD 12.2) years, almost entirely women (29/30, 98%), working in an outpatient practice (29/30, 98%), and mainly having a medium school education (21/30, 68%). Nurses from all 4 regions were included with a range of 9 (30%) nurses from northern and 5 (16.7%) nurses from southern Germany. Patients (n=34) had a mean age of 47.7 (SD 16.8) years with a range between 20 and 77 years, 47% (16/34) were female. The majority of patients had a high school education (24/34, 71%) and were from northern federal states (29/34, 85%). Patients from eastern Germany did not participate. Each targeted indication was covered. The indications hidradenitis suppurativa, atopic dermatitis, and acne were represented by 4 participants, respectively. Psoriasis was represented by 10, chronic wounds by 6, and skin cancer by 5 participants (Table 1).

**Table 1:** Demographic characteristics of participants.

			Dermatologists (n=30)		Nurses (n=30)		Patients (n=34)		
**Range of participants per focus group**									
	Minimum	5		4		5			
	Maximum	6		7		6			
**Age (years)**									
	Minimum	34		23		20			
	Maximum	69		60		77			
	Mean (SD)	51.3 (8.4)		37.5 (12.2)		47.7 (16.8)			
Female participants, n (%)			12 (40)		29 (98)		16 (47)		
**Regional variation, n (%)**									
	West	12 (40)		7 (23.3)		3 (8.8)			
	North	6 (20)		9 (30)		29 (85.3)			
	South	7 (23)		5 (17)		2 (6)			
	East	5 (17)		7 (23)		0 (0)			
Rural area, n (%)			7 (23)		10 (33)		11 (32)		
**Health care sector, n (%)**									
	Outpatient practices	23 (77)		29 (98)		—^a^			
	Outpatient clinic	1 (3.3)		1 (3.3)		—			
	Both	6 (20)		0 (0)		—			
**School education, n (%)**									
	Low	—		0 (0)		2 (3)			
	Medium	—		21 (68)		8 (23)			
	High	—		9 (32)		24 (71)			
**Use of digital devices at least once a week, n (%)**									
	PC	29 (97)		28 (93)		30 (88)			
	Tablet	15 (50)		15 (50)		15 (44)			
	Smartwatch	6 (20)		8 (27)		6 (18)			
	Smartphone	30 (100)		30 (100)		33 (97)			
**Use of digital apps, n (%)**									
	Search engine	30 (100)		30 (100)		34 (100)			
	Social media	13 (43)		24 (80)		23 (68)			
	Instant messenger	29 (97)		30 (100)		32 (94)			
	Podcasts	16 (53)		13 (43)		10 (30)			
	Videos (eg, YouTube or Netflix)	29 (97)		21 (70)		31 (91)			
	Encyclopedia (eg, Wikipedia)	28 (93)		19 (63)		28 (82)			
	Online banking	27 (90)		26 (87)		30 (88)			
	Sports and fitness apps (eg, Strava or Garmin)	14 (47)		18 (60)		23 (70)			
	Use of DHIs^b^, yes	17 (57)		14 (47)		2 (6)			
	Recommendation of DHIs, yes	17 (57)		9 (33)		2 (6)			
**Indications, n (%)**									
	Psoriasis	—		—		11 (32)			
	Hidradenitis suppurativa	—		—		4 (12)			
	Chronic wounds	—		—		6 (15)			
	Atopic dermatitis	—		—		4 (12)			
	Acne	—		—		4 (12)			
	Skin cancer	—		—		5 (18)			

^a^Not applicable.

^b^DHI: digital health intervention.

Nearly every participant of each stakeholder group used a personal computer or smartphone at least once a week and digital apps such as online banking, instant messengers, and search engines (Table 1). Every second dermatologist had used or recommended DHI within the last 12 months before participating in the focus groups (17/30; 57%). Among nurses, 47% (14/30) worked in practices that had used DHIs within the last 12 months, but only a minority of patients had any experiences with DHIs (2/34; 6%).

### Categories of the Focus Groups

The following section describes all deductively used constructs and inductively identified main and subcategories. For some subcategories, we provide representative quotes. All constructs (A-E), main categories, and subcategories are presented in Tables 2-6. Further, 1 representative quote per subcategory is presented in Multimedia Appendix 3. The letters and numbers in front of the quotes indicate the focus group (G1 to G6) and participant number in the respective stakeholder group (P1 to P6: patient, D1 to D6: dermatologist, and N1 to N6: nurse).

**Table 2:** Construct A: attitude toward technology.

Main and subcategories			Physicians		Nurse		Patients
**Positive**							
	Interest in using digital health interventions	—^a^		—		✓^b^	
	Digitalization in the medical field is deemed necessary	—		✓		—	
	Dermatologists are required to participate in digitalization to have a voice in shaping the system	✓		—		—	
	Higher acceptability among younger patients	✓		—		—	
	Acceptability of digital health intervention if used complementary to in-person consultation	✓		✓		✓	
**Negative**							
	Not willing to switch dermatologists for lack of offering digital health interventions	—		—		✓	
	Fast pace of digitalization makes life more difficult	✓		—		—	
	Fear of being replaced by digital health intervention	✓		—		—	
	Older patients prefer personal consultation	—		✓		—	
	Nurses prefer personal consultation	—		✓		—	
	Fear of data misuse by third parties	✓		✓		✓	
**Neutral**							
	Patients are unrestrained toward their data privacy	✓		—		—	
	Economic concerns are important for decision-making	—		✓		—	
	Physicians rate personal impressions over evidence	✓		—		—	
	The dermatologist sees no need for adjustments	✓		—		—	

^a^“—”: statement related to this subcategory did not occur for this group.

^b^“✓”: statement related to this subcategory did occur in this group.

**Table 3:** Construct B: performance expectancy.

Main and subcategories		Physicians	Nurse	Patients
**Positive**				
	Greater involvement of patient in treatment	✓^a^	✓	✓
	Improvement of patient-physician relationship	✓	—^b^	✓
	Promotion of need-based care	✓	—	—
	Promotion of standardized care	✓	—	—
	Improvement of follow-up consultations	—	—	✓
	Support of treatment process through digitized patient data	—	✓	—
	Reduction of unnecessary travel to medical appointments	—	—	✓
	Time savings during the treatment process	—	✓	—
	Usefulness of data for research purposes	—	—	✓
**Negative**				
	Impersonal patient-physician relationship	✓	✓	✓
	Additional workload	✓	✓	—
	Overload of information	✓	—	—
**Requirement**				
	Technical functionality should result in an added value	✓	^—^	^—^
	Additional value for both patient and physician	✓	✓	✓

^a^“✓”: statement related to this subcategory did occur in this group.

^b^“—”: statement related to this subcategory did not occur for this group.

**Table 4:** Construct C: effort expectancy.

Main and subcategories		Physicians	Nurse	Patients
**Positive**				
	High digital competencies among nurses	✓^a^	—^b^	—
	High digital competencies among younger patients	—	✓	—
	Decreasing proportion of patients with low digital competencies over time	—	✓	—
**Negative**				
	Low digital competencies among older patients	✓	✓	✓
	Difficulties in assessing the integrity of apps	✓	—	—
	Low digital competencies among nurses	✓	—	—
	Initial high effort to implement digital health interventions	—	✓	—
	Low digital competencies among older physicians	—	✓	—
	Exclusion of digital illiterate patient groups from care	✓	—	—
**Requirements**				
	Easy-to-use apps	✓	✓	—
	Easy integration into daily routines	—	✓	—

^a^“✓”: statement related to this subcategory did occur in this group.

^b^“—”: statement related to this subcategory did not occur for this group.

**Table 5:** Construct D: social influence.

Main and subcategories		Physicians	Nurse	Patients
**Positive**				
	Trust in physicians’ recommendations	—^a^	✓^b^	✓
	Trust in colleagues’ recommendations	✓	✓	—
	Physicians rate colleagues’ recommendations over evidence	✓	—	—
	Trust in recommendations or digital health developments of or by trustworthy institutions	—	—	✓
	Trust in recommendations of physician associations	✓	—	—
**Negative**				
	Dependence on physicians’ acceptance	—	✓	✓
	Dependence on patients’ acceptance	✓	—	—
	No trust in nurses’ recommendations by patients	—	✓	—

^a^“—”: statement related to this subcategory did not occur for this group.

^b^“✓”: statement related to this subcategory did occur in this group.

**Table 6:** Construct E: facilitating conditions.

Main and subcategories		Physicians	Nurse	Patients
**Facilitators**				
	A single app for different purposes	—^a^	✓^b^	✓
	A single app for the same purpose used by all physicians	✓	—	—
	Clear data access permissions	—	—	✓
	Possibility to choose between analog and digital health intervention	—	—	✓
	Engagement of nurses in digital processes	—	✓	—
	Patients possess digital devices	—	✓	—
	General trust in data protection and security	✓	—	—
	Sufficient reimbursement	✓	✓	✓
	Pandemic has accelerated the progress of digitalization	—	✓	—
**Barriers**				
	Difficult to integrate digital health interventions into busy daily routines	✓	✓	—
	Use of outdated technology in practices	—	—	✓
	High-maintenance of IT infrastructure	✓	—	—
	Current data protection regulations impede the functionality of digital health interventions	✓	—	✓
	Insecurity toward data privacy laws	✓	—	—
**Neutral**				
	Data privacy is very important in medical practices	—	✓	—

^a^“—”: statement related to this subcategory did not occur for this group.

^b^“✓”: statement related to this subcategory did occur in this group.

### Attitude Toward Technology

In general, all groups found DHIs acceptable if they serve as a complement to rather than a replacement of in-person consultations with dermatologists (Table 2):

So I would find such an app good, but it must be complementary to the physicians’ consultation and not that a doctor would say, “Just look on the app,” so to speak, in order to shorten parts of the consultation or the treatment time.

G2, P2

In addition, patients stated a general interest in using DHI in the future but would not change dermatologists for not offering DHIs at their practice (Table 2). Dermatologists also saw acceptability among patients but mainly in younger patients. Nurses emphasized the necessity of digitalization due to the limited availability of time and resources. However, some nurses underscored the preference for in-person consultations from their own and older patients’ perspectives. They additionally highlighted the importance of economic concerns for a decision on a DHI.

All groups expressed a fear of data misuse by third parties, but dermatologists also observed patients having low standards for their own data protection practices.

So patients are self-indulgent when it comes to privacy. Of their own accord. They send you naked photos via WhatsApp. When you get a cell phone, everyone thinks I'm a child pornographer, because there are so many baby bums from vacation with diaper dermatitis on it. So, they are completely, completely uninhibited.

G5, D1

Furthermore, for some dermatologists, their personal impression of a DHI is more important than the scientific evidence. Some dermatologists thought they were required to participate in the digitalization process to have a say in shaping the health care system (Table 2). Other dermatologists expressed pessimism regarding the digitalization process and emphasized how the rapid pace of digitalization makes their work more challenging.

They even voiced concerns about digitalization leading to the replacement of health care professionals.

On the other hand, even as a doctor, you must worry that this digitalization will eventually replace us. For example, we know that rheumatologists and radiologists will soon no longer be able to work properly because artificial intelligence can make much better assessments than radiologists.

G6, D4

### Performance Expectancy

Nurses, dermatologists, and patients collectively mentioned positive performance expectations regarding greater patient engagement in their treatment and care:

But if it goes the other way, as we just discussed that we as patients then take a little more responsibility, and then you can present a condensed summary to the doctor, then maybe it will make sense.

G5, P2

All groups also required that DHIs should result in an additional value for both patients and physicians. On one hand, all 3 stakeholders expected a more impersonal patient-physician relationship whereas on the other hand, an improvement of the relationship was discussed by some patients and dermatologists. Patients mentioned other positive performance expectations such as reduced unnecessary travel, improved follow-up sessions (eg, better-prepared patients and practitioners), and the usefulness of data for additional purposes, such as research. Although dermatologists recognized potential positive impacts of DHIs, such as enabling standardized treatment and promoting need-based care, they stressed the need for technical functionalities to result in added value. For them, existing functionalities did not consistently meet this requirement. Negative effects on dermatologists’ daily work were also anticipated by this group. For instance, they indicated the potential overload of information and additional workload associated with DHIs:

So I think, that will not be a relief. There will be additional work. For example, most of the patients with a video consultation, must come into the office afterwards [...]. So, it costs more time and maybe you could do it in the evening when you really need to relax. I already have an (exhausting) day anyway, and then a video consultation in the evening?

G3, D6

This additional workload was also mentioned by some nurses. Other nurses noted the potential for time savings for both physicians and patients:

... when it’s digital, the doctor can immediately write in the medical history, I can prepare the prescription, the doctor is sitting in the treatment room, I’m sitting at the reception. He writes it in the medical history, and I can prepare it at the same time, so ... For the patient, too, it’s much, much, much shorter in terms of time.

G5, N5

The potential of digitized patient data to support a patient’s treatment process, for example through optimized therapy decisions, was also identified by nurses.

### Effort Expectancy

All 3 stakeholder groups articulated the existence of low digital competencies among older patients. Dermatologists even concluded that digitalization would result in the exclusion of digitally illiterate patient groups from care (Table 4):

In my opinion, 20-30 percent of humanity is still digitally illiterate. That includes people like my mother, who somehow managed to crash the Internet I think three times by now. Not the computer, the Internet.

G6, D2

Some dermatologists believe that nurses possess high digital competencies which are crucial for implementing DHIs, while others perceive nurses to have low digital competencies. Nurses, in turn, observed that physicians also exhibited low digital competencies. Another barrier to the adoption of DHIs, as stated by some dermatologists, is the challenge of assessing the credibility and trustworthiness of apps. Nurses described the high effort that is required to adapt to a DHI in a practice, yet they emphasized the considerable advantage once the DHI is successfully implemented:

As with everything that is new at first, it is of course a lot of work, a huge amount of work for those involved, but then I think there is also a great benefit underneath. [...]at the beginning, of course, it was new and incredibly difficult, and everyone said, “oh God, oh God, oh God,” and now everyone expresses their gratitude, saying, “thank God, we have it better now.”

G6, N6

According to nurses, DHIs should be easy to integrate into daily routines. Dermatologists and nurses agreed on the importance of ease of use for DHIs.

### Social Influence

Social influence played a role in discussing DHIs for all stakeholder groups, especially regarding trust (Table 5). Dermatologists expressed their trust in recommendations from their physician associations as well as colleagues. Dermatologists even emphasized trusting the opinions of their colleagues over scientific evidence:

The most important evidence is still colleagues you trust and who also have experience, and there you listen to three, four opinions, and if that goes in one direction, then you try that.

G2, D5

Patients place their trust in the opinions of trustworthy institutions (eg, patient organizations) and dermatologists. Patients and nurses shared the viewpoint that usage of DHI is highly dependent on dermatologists’ acceptance:

The physicians [...] play the main role. Because with him is the main interaction and he is the main person to whom one would turn, be it digital or otherwise. So, if the- if the doctor rejects digitalization, then there’s no point in any of this. So, then it’s a side event.

G2, P2

This is supported by patients’ lack of reliance on nurses’ recommendations:

It doesn't matter if it's a recommendation for something, if it's just a cream for something, if it's something I say at the front, it's only something she says. But ...uh... if the doctor says it in the treatment room, then, then it's great and then it's like that and then we'll definitely buy it.

G3, N2

### Facilitating Conditions

Sufficient reimbursement of DHIs emerged as an important facilitator for all stakeholder groups. As additional facilitators patients and nurses stated that they would like to have a single app for multiple purposes:

The described applications are all scattered pieces. I can make a constellation here, I can get a second opinion here, I can at best download my prescription to my cell phone instead of in paper form. Um, I have to lug doctor’s letters from A to B, then there’s faxing, so I would prefer an integrated solution, whether that’s for dermatology or anything else.

G1, P3

Patients identified technical barriers as hindrances to the implementation of DHIs such as outdated technology in medical practices (Table 6). They also emphasized the importance of clear access permissions to transfer data between physicians. Nurses were willing to participate in digital processes to support an easy integration of DHIs in their practices:

I would have liked to offer video consultation hours. You can also really do it in such a way that a doctor is present, a nurse is present, and that all the other things, i.e., writing and doing, i.e., writing materials, etc., are taken over by the nurse, that's not a problem. And so that the communication of doctor and patient takes place, that could have worked. But it shouldn't be. So yes, too bad.

G3, N3

The nurses positively pointed out that nearly all patients possess digital devices, and they described how the pandemic has accelerated the digitalization in their practices. Dermatologists preferred a single, standardized app for a specific purpose that would be used by all physicians instead of several platforms with diverse accounts and handling. They identified the considerable effort required for the IT infrastructure as a key barrier to the implementation of DHIs. Additionally, both dermatologists and nurses highlighted the challenge of integrating DHIs into their already demanding daily workloads in medical practices:

Well, I haven’t done it [store-and-forward telemedicine] either. I won’t do it either. But that’s usually because of the time factor. If you have so many functions in addition to your work in the practice, then you think: Do I still sit down there in the evening and answer something like that?

G5, D2

Nurses generally describe that data privacy is very important in medical practices. Some dermatologists generally trust whereas others feel insecure about data protection and security. Moreover, dermatologists highlighted the negative effect of current data regulations on the functionality of DHIs.

## Discussion

### Principal Findings

The current use of DHIs in Germany, both in general and dermatological care, remains low. Therefore, we conducted a qualitative focus group study to explore and exploratively compare patients’, nurses’, and dermatologists’ attitudes, acceptability, barriers, and facilitators on the implementation of DHIs in dermatology. Additionally, we exploratively compared their perspectives. Patients and nurses had a generally positive stance and optimistic attitude toward digitalization and assumed largely positive performance expectancies. In contrast, dermatologists showed more different opinions with some expressing positive performance expectations, while others anticipated increased workload and information overload with the implementation of DHIs. While sufficient reimbursement and patients owning digital devices were identified facilitators, insecurity regarding data privacy laws and the difficulty of integrating DHIs into an already busy day were identified barriers.

Although our research identified a more negative stance of dermatologists toward DHIs in comparison to patients and nurses, quantitative research yielded mixed results [23,39]. It is important to consider that the acceptability of DHIs is influenced by the context in which they are used. For instance, willingness to use teledermatology is lower for severe and acute conditions compared to minor problems [39].

Independent of the context, dermatologists’ acceptability is crucial for actual usage, as patients and nurses also pointed out, and can mitigate barriers, including low patient demand, problems with the technology, and lack of financial resources [33,40]. The influential role of dermatologists stems from their function as gatekeepers in introducing medical innovations, including DHIs, into care. Additionally, their positive stand on a DHI can signal credibility to patients, nurses, and colleagues. The low acceptability by dermatologists seems to be a barrier to the implementation of many DHIs. However, improving physicians’ acceptability could become a facilitator, particularly as they trust their colleagues’ recommendations and patients trust their health care providers. To increase acceptability among physicians, reimbursements should be clarified, and patient benefits should be aligned with an added value for dermatologists as proposed by all stakeholder groups in our study and as found in the literature [41].

In recent years, physicians were required to make financial investments in the IT infrastructure without a clear perceived benefit leading to hesitance in adopting new DHIs in clinical practice [42,43]. Both, the missing positive financial perspective and missing benefits are established barriers to the introduction of eHealth interventions [22]. While 98% of outpatient medical practices are connected to the nationwide telematic infrastructure, only a minority are satisfied with its services [44]. Other countries, especially Estonia and Canada were more successful in establishing an eHealth-infrastructure that allowed a fast uptake of DHI services by physicians and other health care providers [45,46]. In addition, physicians in both countries were financially incentivized to adopt DHIs [45,46].

The infrastructure in Germany is therefore considered a barrier to the seamless adoption of many DHIs [41]. This resonates with the cautious perspectives of dermatologists on DHIs in our study. In addition, the economic and resource advantages for society, health insurance, or patients, such as reduced follow-up appointments or travel costs, may not necessarily translate into added value for dermatologists [47]. These concerns should be addressed so that physicians are more likely to adopt and recommend new technologies [48].

Dermatologists and nurses expressed concerns about an increased workload. Other nurses also identified time savings as an effect of DHIs. Both perspectives were identified across other medical fields [22]. The potential increased workload for dermatologists may be explained by the DHIs not aligning with working routines or dermatologists needing additional time for patient care, such as explaining DHI to patients [49]. From another angle, even successfully implemented DHIs may result in additional workload as technological progress enables faster completion of tasks (eg, accelerated patient consultation per store-and-forward teledermatology), but increases in the number of tasks (eg, more patients per day). Consequently, actual time being spent inactive is reduced and time pressure is amplified (theory of social acceleration) [50]. Yet, the ability to complete tasks faster may also result in a reduced workload for dermatologists and nurses.

The true impact of DHIs on the patient-physician relationship remains uncertain and will largely depend on the extent and the specific context in which the DHI is introduced [51]. Following the social acceleration theory, using DHIs can result in increased but less relationship-building communication between dermatologists and patients [50]. Time savings, possibly leading to more available time for individual patients, can also foster a trusting patient-physician relationship.

An identified barrier to using DHIs in dermatology is the lack of digital competencies and knowledge among patients, practitioners, and nurses. Although internet use and competencies have increased in the last decade [52,53], 1 recent European survey estimated that 22% (Norway) to 58% (Germany) of Europeans have inadequate digital health literacy levels. Among older and less educated individuals, the percentage is even higher [54]. To avoid the exclusion of patient groups (digital divide) [55], participants in our study even emphasized the need for analog alternatives to DHIs. To increase adoption rates, digital health literacy skills need to be improved and services must be adapted to the digital competency levels of intended users and should always be easy to use [56]. For physicians, knowledge of DHIs and their evidence base should be incorporated into medical curricula and continued medical education [57].

Participants in our study discussed data privacy and security from different perspectives. For some, data privacy risk was a reason for the nonusage of DHIs, consistent with findings in the literature [58]. Conversely, others noted that the enforcement of data privacy laws hindered the development of effective DHIs. This might not be generalizable to other countries, as German citizens have generally stronger concerns regarding data privacy and protection [59,60].

Furthermore, dermatologists complained about the considerable maintenance burden of the IT infrastructure, partially driven by data privacy regulations. Increasing IT costs and dissatisfaction with IT were also identified in the literature [43,61]. The difficulties assessing the integrity of DHIs may be explained by the missing transparency of data privacy policies of many DHIs [62]. Health data are one of the most sensitive data requiring an enforced data privacy regulation. However, the enforcement of data security policies should be balanced in the sense that data are protected while the usage of the app remains convenient and useful. Other European countries under the same regulatory framework, including Estonia, seem to have achieved this balance [45].

While our study provided valuable insights into the attitudes toward DHIs from patients’, dermatologists’, and nurses’ perspectives, it is important to acknowledge several limitations when interpreting our findings. The digital conduction of our focus groups may have excluded individuals with limited or no digital competencies. To at least mitigate this limitation, we followed the STEER recommendations by conducting test calls to enable individuals with limited competencies to participate [29]. Furthermore, we established ground rules to ensure a comfortable and private setting for all participants [29]. Thereby, we also ensured a smooth discussion. The possibility of social desirability cannot be completely excluded, but it may have been low due to the private setting, ensured confidentiality, and anonymity of the focus groups, as well as the nonsensitive topic of digital health [63]. Moreover, participants with a digital background or interest in the topic may have been more motivated to join the web-based focus groups. Despite efforts to recruit a diverse range of participants through purposeful sampling [42], it should be noted that the majority of patients in our study were well-educated. Additionally, it is important to mention that the apps discussed in our study were hypothetical limiting participants’ ability to fully assess the practical implications of using these interventions in real-life scenarios, as only a minority of patients had actual experience with a DHI. Considering all limitations, including the general qualitative nature of this study and the fact that it was carried out in Germany only, the results may not be completely generalizable to other medical fields and health care systems. However, as shown, many aspects are also described in the international literature and the findings may, therefore, be relevant to a wider audience.

### Conclusions

To ensure a successful digitalization process in dermatology, it is essential to develop easy-to-use apps that bring additional value to all stakeholders involved. Dermatologists’ acceptance is crucial as dermatologists can serve as a facilitator in their role. Incorporating their perspectives during the development phase can help align future digital interventions with clinical practices, increasing acceptance and usage.

Due to the lack of digital health literacy among the population DHIs should be designed to accommodate different levels. Analog access options should be provided to prevent the exclusion of less digitally literate patient groups in the near future.

Data privacy and security concerns must be taken seriously, as they are crucial for maintaining trust in digital interventions. They can function as barriers to interventions’ effectiveness and cause users’ insecurities. Successful digitalization in dermatology requires striking a balance on data privacy to allow for the development of effective interventions.

In summary, our findings can aid researchers, developers, and decision makers in comprehending diverse stakeholder perspectives. This can help create successful DHIs and subsequent implementation strategies, thereby enhancing the acceptability and uptake of DHIs.

## Appendix

Multimedia Appendix 1 COREQ (consolidated criteria for reporting qualitative research) checklist.

Multimedia Appendix 2 Semistructured interview guide (German version and English translation).

Multimedia Appendix 3 Overview of categories per stakeholder group and representative quote for the category.

## Abbreviations

COREQ: consolidated criteria for reporting qualitative research
DHI: digital health intervention
STEER: Stability of Group Number, Technology, Environment, Evaluation, and Recruitment
UKE: University Medical Center Hamburg-Eppendorf
UTAUT: unified theory of acceptance and use of technology
