# Supplementary material for: Exploring Acceptability, Barriers, and Facilitators for Digital Health in Dermatology: Qualitative Focus Groups With Dermatologists, Nurses, and Patients
Source: JMIR Dermatol. 2024 Sep 3;7:e57172. doi: 10.2196/57172 (PMC11408893; doi:10.2196/57172)
Supplement: Multimedia Appendix 2 [file derma_v7i1e57172_app2.docx]

**Semistructured interview guide (German version and English translation)**

**Original German Version**

**Information:** This is the patient version of the semi-structured interview guide. The version for nurses and dermatologists is closely aligned and is available upon request.

**Leitfaden für Fokusgruppen zur Erfassung der Erfahrungen, Bedarfe, Akzeptanz sowie Barrieren und fördernden Faktoren beim Einsatz digitaler Anwendungen**

**in der Dermatologie aus Sicht von PatientInnen**

Begriffserläuterungen

(Digitale) Anwendungen Smartphone- oder Tablet-Anwendungen, Apps und Internetportale bzw. Webseiten

(Digitale) Gesundheitsanwendungen Jegliche (digitale) Anwendungen, welche zur direkten Diagnosestellung, Überwachung oder Behandlung der Erkrankung bzw. zur Diagnosestellung, Überwachung oder Behandlung von Begleiterkrankungen eingesetzt werden können.

**Einführung in die Fokusgruppe**

Ich freue mich sehr, dass Sie sich bereit erklärt haben an der Fokusgruppe teilzunehmen.

*Ich stelle mich vor.* Sie als Teilnehmende müssen sich nicht vorstellen, da die Teilnahme anonym ist. Wenn Sie sich gegenseitig ansprechen möchten, dann nutzen Sie einfach den Namen oder die Abkürzung, die im WebEx-System eingetragen wurde. Sie sind alle ausgewählt worden, da Sie an mindestens einer chronischen Hauterkrankung leiden.

*Erläuterung der Studieninformation und Fragen beantworten (explizit nach Fragen fragen).*

Vorab: Die Teilnahme ist freiwillig. Wenn Sie zu irgendeinem Zeitpunkt das Interview abbrechen oder eine Pause machen wollen, sagen Sie mir einfach Bescheid. Natürlich können Sie auch eine Frage mal nicht beantworten, wenn Sie nicht möchten. Sie können auch immer Fragen stellen. Fühlen Sie sich bitte auf jeden Fall frei sich zu äußern und Ihre Meinung zu sagen, denn mich interessieren insbesondere unterschiedliche Meinungen. Es geht heute nicht darum einen Konsens zu finden. Und deshalb gibt es keine richtigen oder falschen Antworten, denn es geht um Ihre Meinung und Ihre Erfahrungen. Ich bitte Sie somit ganz frei zu antworten.

Wir werden die Diskussion auf Tonband aufnehmen, damit uns keine Informationen verloren gehen.

Wenn Sie keine weiteren Fragen haben, können wir direkt mit dem Interview beginnen.

1. **Bedarfe von digitalen Anwendungen in der Dermatologie**

Zu Beginn möchten wir gerne über Ihre Bedarfe bei der Versorgung ihrer Erkrankung sprechen.

- 1. Inwieweit haben Sie sich schon mit anderen Personen über Probleme mit der Behandlung oder Probleme mit der Versorgung Ihrer Erkrankung ausgetauscht?
     - - Austausch in real oder über Patientenforen im Internet
       - Haben Sie Gespräche mit Familienmitgliedern oder FreundInnen geführt?
     1. Welche (weiteren) Themen haben Sie dabei besprochen oder diskutiert?
        - fehlende Informationen vom Arzt
        - fehlende Kommunikation mit Arzt
        - Schwierigkeit einen Termin zu finden
        - Unzufriedenheit mit derzeitiger Therapie
        - Einfluss von Erkrankung auf die Psyche
        - Häufigkeit von Arztterminen
        - keine Chance Fragen zu stellen
  2. Inwiefern sind Sie zufrieden mit der Versorgung durch Ihre ÄrztInnen?
     1. Was ist eher positiv in der Versorgung?
     2. Was ist weniger zufriedenstellend in der Versorgung?
     3. Was fehlt für ein perfektes ÄrztInnen-PatientInnen-Gespräch?
     4. Inwiefern sind Sie zufrieden mit der Kommunikation mit Ihrem Arzt?
     5. Inwiefern sind Sie zufrieden mit den Abläufen in Ihrer Versorgung?
  3. Inwieweit gibt es Themen, bei denen Sie konkret Hilfe benötigen, aber keine erhalten bzw. nur schwer erhalten?
     - - Organisation des Behandlungsplans
       - Wissenslücken

1. **Potenzieller Nutzen und Präferenzen von digitalen Anwendungen**

Nun wollen wir konkreter über digitale Gesundheitsanwendungen sprechen. Wir haben uns vorab ja schon ein paar Informationen von Ihnen eingeholt, um zu sehen, wie viel Erfahrungen Sie bereits damit gesammelt haben:
*nahezu alle/einige/wenige Personen in der Gruppe ein Smartphone oder Tablet besitzen und die alle/meisten/einige von Ihnen gängige Anwendungen, wie Soziale Netzwerke, nutzen und alle/meisten/einige diese auch für Fragestellungen rund um Ihre Gesundheit nutzen. Die Gruppe ist also gut durmischt/diesbezüglich nahezu einheitlich.*

Wenn wir von digitalen Gesundheitsanwendungen sprechen, meinen wir Apps, Internetportale, Online-Anwendungen oder Wearables (z.B. Smartwatches) für Ihr Smartphone, Tablet oder Computer, die Sie oder Ihren Arzt bei der Diagnosestellung, Überwachung oder Behandlung Ihrer dermatologischen Erkrankung oder einer Begleiterkrankung unterstützen.

- 1. Haben Sie zu dieser Definition eine Frage?

Ich stelle Ihnen nun mehrere digitale Gesundheitsanwendungen für die Dermatologie vor, um diese gemeinsam mit Ihnen zu diskutieren. Einige Anwendungen gibt es, andere haben wir ggf. auch angepasst – es sind somit potenzielle Anwendungen, von uns einfach beispielhaft zusammengestellt. Wir haben uns dabei an Beispielen aus der wissenschaftlichen Literatur orientiert.

Anwendung 1: Medikations-Tracker

Stellen Sie sich eine digitale Anwendung vor, welche Sie dabei unterstützt ihren Behandlungs- oder Medikationsplan zu befolgen. Die Anwendung erinnert Sie an das Auftragen einer Salbe oder die regelmäßige Einnahme von Tabletten, indem Sie mit einer Nachricht daran erinnert werden (z.B. 3-mal am Tag). Die Einnahme der Medikation kann dann über eine Taste in der Anwendung bestätigt werden. Manchmal können Sie z.B. zusätzlich gebeten werden ein Foto vom Auftragen der Salbe mitzusenden. Es konnte bereits nachgewiesen werden, dass PatientInnen sich damit eher an den Behandlungsplan der ÄrztInnen halten.

- 1. Inwiefern können Sie sich vorstellen eine solche Anwendung für ihren Behandlungsalltag zu nutzen?
     1. Welche zusätzlichen Funktionen/Standards müsste diese Anwendungen haben, damit Sie diese regelmäßig nutzen.
     2. Was könnte Sie gegebenenfalls davon abbringen eine solche Anwendungen zu nutzen?
        - Zeit
        - zu kompliziert bzw. aufwändig

Anwendung 2: PatientInnen-Monitoring

Stellen Sie sich vor es gäbe eine Anwendung, die alle wichtigen Daten über Ihre Behandlung und Erkrankung festhält und so eine Verlaufskontrolle ermöglicht. Sie als Patient können viele wichtige Informationen eintragen. Zum Beispiel können Sie regelmäßig Fragen zu Ihrer Lebensqualität oder Zufriedenheit mit der derzeitigen Behandlung beantworten, ein Symptomtagebuch führen, Fotos oder Inhalte aus anderen Anwendungen hochladen (z.B. aus Fitnesstrackern, Schrittzählern, Schlaftrackern). Sie können sich die Eintragungen dann jederzeit anschauen, auch im Zeitverlauf, und Sie könnten die Daten mit Ihrem Arzt besprechen oder sie ihm zur Verfügung stellen.

- 1. Inwiefern können Sie sich vorstellen eine solche Anwendung für ihren Behandlungsalltag zu nutzen?
     1. Welche zusätzlichen Funktionen/Standards müsste diese Anwendung haben, damit Sie diese regelmäßig nutzen?
     2. Was könnte Sie gegebenenfalls davon abringen eine solche Anwendungen zu nutzen?
     3. Inwiefern können Sie sich vorstellen solche Daten in einem ÄrztInnen-PatientInnen-Gespräch zu nutzen?

Stellen Sie sich vor, dass Ihr/e Arzt/Ärztin zusätzlich Informationen in diese Anwendung eintragen oder hochladen könnte, wie z.B. die derzeitige Medikation oder klinische Daten, wie z.B. den Schweregrad der Erkrankung. Die Daten des/r Arztes/Ärztin könnten dann mit den von Ihnen eingegebenen Daten zusammengeführt werden und Sie hätten eine noch ausführlichere Verlaufskontrolle.

- 1. Inwiefern können Sie sich vorstellen eine solche Anwendung für ihren Behandlungsalltag zu nutzen?
     1. Inwiefern können Sie sich vorstellen solche Daten in einem ÄrztInnen-PatientInnen-Gespräch zu nutzen?

Anwendung 3: Digitale Kommunikation mit ÄrztInnen
Stellen Sie sich eine digitale Anwendung vor, mit welcher Sie die Möglichkeit haben, Ihrem/Ihrer Arzt/Ärztin schriftlich konkrete Fragen zu Laborwerten, der Therapieform oder zu auftretenden Symptomen zu stellen - ähnlich einer E-Mail oder einem Instant Messenger, wie WhatsApp/Telegram/Threema. Der Arzt/die Ärztin würde in einem bestimmten Zeitrahmen antworten.

- 1. Inwiefern können Sie sich vorstellen eine solche Anwendung für Ihren Behandlungsalltag zu nutzen?
     1. Was könnte Sie gegebenenfalls davon abringen eine solche Anwendung zu nutzen?
     2. Welche zusätzlichen Funktionen/Standards müsste diese Anwendung haben, damit Sie diese regelmäßig nutzen.
     3. Welche (weiteren) Situation muss gegeben sein, damit Sie eine Videokonferenz nutzen?
  2. Inwiefern wäre die Speicherung der Daten oder der Zugriff auf die Daten durch andere relevant für Ihre Nutzung der Anwendung?

Immer etablierter wird die Möglichkeit der Videosprechstunde. Diese ermöglicht Ihnen den Arzttermin via Videokonferenz wahrzunehmen. Sie benötigen für die Besprechung lediglich ein internetfähiges Gerät.

- 1. Inwiefern können Sie sich vorstellen eine solche Anwendung für Ihren Behandlungsalltag zu nutzen?
     1. Was könnte Sie gegebenenfalls davon abringen eine solche Anwendung zu nutzen?
     2. Welche zusätzlichen Funktionen/Standards müsste diese Anwendung haben, damit Sie diese regelmäßig nutzen.

Stellen Sie sich nun eine digitale Anwendung vor, mit der Sie einen weiteren bzw. einen Ihnen nicht bekannte/n DermatologIn kontaktieren können, z.B. zur Zweitmeinung oder für andere dermatologische Beschwerden. Dabei können Sie Bilder von Ihrem Hautproblem in eine App oder Online-Anwendung hochladen, einen kurzen Fragebogen zu Symptomen, der Erkrankung oder der derzeitigen Behandlung ausfüllen und Ihre Beschwerden schriftlich beschreiben. Ein/e DermatologIn begutachtet dann Ihren Fall, stellt bei Bedarf Rückfragen und gibt Ihnen innerhalb von 48 oder 72 Stunden eine fachärztliche Einschätzung - entweder eine Empfehlung für die Behandlung inkl. Rezept oder eine Überweisung. Sie erhalten ebenfalls einen Arztbrief.

a) Inwiefern können Sie sich vorstellen eine solche Anwendung zu nutzen?

- - - Zweitmeinung
    - Urlaub
    - Etwas Akutes
    1. Was könnte Sie gegebenenfalls davon abhalten eine solche Anwendung zu nutzen?
    2. Welche zusätzlichen Funktionen/Standards müsste diese Anwendung haben, damit Sie diese regelmäßig nutzen?
  1. Inwiefern können Sie sich vorstellen eine solche Anwendung zu nutzen, wenn Sie die Konsultation selbst zahlen müssen?

Anwendung 4: Selbstmanagement und Aufklärung

Stellen Sie sich eine digitale Anwendung vor, die Ihnen Informationen über Ihre Erkrankung und Therapien zur Verfügung stellt, darunter auch konkrete Tipps und Anleitungen zum besseren Umgang mit Ihrer Erkrankung im Alltag, z.B. verständliche Informationen zur Diagnose und Therapien oder Tipps zum Umgang mit Stress und psychischen Beeinträchtigungen durch Ihre Erkrankung. Dies kann mithilfe von Texten, Videos oder Bildern stattfinden.

1. Inwiefern können Sie sich vorstellen eine solche Anwendung zu nutzen?
   - 1. Was könnte Sie gegebenenfalls davon abringen eine solche Anwendung zu nutzen?
     2. Welche zusätzlichen Funktionen/Standards müsste diese Anwendung haben, damit Sie diese regelmäßig nutzen?
   1. Welche der genannten Themen fänden Sie besonders interessant bzw. welche Themen wären zusätzlich für Sie interessant?

Trade-OFF aller Anwendungen

Leider ist es nicht möglich alle Anwendungen gleichzeitig zu entwickeln.

- 1. Daher würden wir sehr gerne von Ihnen wissen, welche dieser Anwendungen Sie priorisieren würden. Könnten Sie ggf. erläutern, warum Ihnen die jeweilige Anwendung besonders wichtig ist? Zur Erinnerung: folgende Anwendungen wurden vorgestellt und diskutiert:
     - - Medikations-Tracker
       - PatientInnen-Monitoring
       - Digitale Kommunikation mit ÄrztInnen
       - Selbstmanagement und Aufklärung

Arzt-PatientInnen-Interaktion

Nachdem wir viele Anwendungen diskutiert haben:

- 1. Was glauben Sie: Inwiefern kann der Einsatz von digitalen Anwendungen Ihre Beziehung oder Kommunikation zum Hautarzt verändern?

1. **Akzeptanz von digitalen Gesundheitsanwendungen**

Nachdem Sie nun unsere Beispiele kennengelernt haben, aber ggf. auch andere digitale Gesundheitsanwendungen kennen:

- 1. Wie stehen Sie zur Digitalisierung im Gesundheitswesen allgemein?
     - - Positiv aufgeschlossen, da Sie z.B. schneller in Kontakt mit ÄrztInnen treten können
       - Negativ, da Ihnen der Datenschutz bei solchen Anwendungen Sorgen macht
  2. Welcher Nachweis über die Qualität und den Nutzen der Anwendung muss vorliegen, damit Sie diese nutzen?
     - - Gute Bewertung im App Store / Google Play Store
       - Zertifizierung von TÜV, Stiftung Warentest, Wissenschaftlicher Nachweis
       - Empfehlung von ÄrztInnen
     1. Inwiefern würden Sie sich vorab Informationen einholen? (Online, ÄrztInnen, FreundInnen, andere Betroffene)
     2. Wenn Ihr Arzt Ihnen die Anwendung empfiehlt, würden Sie diese uneingeschränkt nutzen?
  3. Wie bewerten Sie das Thema Datenschutz bei der Nutzung von digitalen Anwendungen?
  4. Wem sollten welche der gesammelten Daten gehören bzw. wo sollten welche Daten gespeichert werden?
- PatientInnen
- ÄrztInnen
- Andere
  1. Inwiefern würden Sie Daten der Forschung zur Verfügung stellen?

1. **Barrieren und fördernde Faktoren für den Einsatz von digitalen Anwendungen**

Denken Sie nochmal an alle digitalen Anwendungen, die Ihnen nun bekannt sind.

- 1. Was könnte Sie persönlich von der Nutzung dieser digitalen Anwendungen abhalten?
     1. Was sind aus Ihrer Sicht Gründe die digitalen Anwendungen nicht zu nutzen?
     2. Was sind die Nachteile von digitalen Anwendungen?
  2. Was könnte Sie persönlich darin bestärken diese digitalen Anwendungen zu nutzen?
     1. Was sind aus Ihrer Sicht Gründe die digitalen Anwendungen zu nutzen?
     2. Was sind die Vorteile von digitalen Anwendungen?

1. **Eigene Ideen und Erfahrungen**
   1. Da Sie der Experte ihrer Erkrankung sind, würden wir zum Schluss gerne von Ihnen wissen, ob Sie selbst eine weitere Idee für Funktionen oder für eine digitale Anwendung haben, welche Sie bei der Versorgung Ihrer Erkrankung unterstützen kann?
      1. Denken Sie an die Themen, die Sie mit anderen PatientInnen, FreundInnen oder der Familie diskutiert haben.
      2. Gibt es Dinge, die Sie heute noch händisch dokumentieren, die sich einfach digitalisieren lassen?
         - Ein Krankentagebuch
   2. Inwiefern gibt es noch digitale Anwendungen/Funktionen, die Sie nutzen, die wir bis jetzt noch nicht besprochen haben?
      1. Was war positiv?
      2. Was war negativ?
   3. Inwieweit können sich alle anderen vorstellen die beschriebene Anwendung für sich zu nutzen?

Gibt es noch irgendwelche Anmerkungen oder Fragen Ihrerseits?

Vielen herzlichen Dank für Ihre Teilnahme!

**English Translation**

**Information:** This is the patient version of the semi-structured interview guide. The version for nurses and dermatologists is closely aligned and is available upon request.

**Semi-structured interview guide for focus groups to explore experiences, needs, acceptance, barriers, and facilitating factors in the use of digital applications in dermatology from the patient's perspective**

Definitions

(Digital) Application Smartphone or tablet applications, apps and internet portals or websites

(Digital) Health Intervention Any (digital) applications that can be used for the direct diagnosis, monitoring, or treatment of the disease or for the diagnosis, monitoring, or treatment of concomitant diseases.

**Introduction to the focus group**

I am very pleased that you have agreed to take part in the focus group. Let me introduce myself. As a participant, you do not need to introduce yourself, as participation is anonymous. If you would like to address each other, simply use the name or abbreviation entered in the WebEx system. You have all been selected because you are diagnosed with at least one chronic skin disease.

*Explain the study information and answer questions (explicitly ask for questions).*

First of all: Participation is voluntary. If at any time you want to stop the interview or take a break, just let me know. Of course, you can also choose not to answer a question if you do not want to. You can always ask questions. In any case, please feel free to express yourself and give your opinion, because I am particularly interested in different opinions. It is not about finding a consensus today. And that is why there are no right or wrong answers, because it is about your opinion and your experiences. I therefore ask you to answer freely.

We will record the discussion on tape so that we do not lose any information.

**1) Needs for digital applications in dermatology**

To begin with, we would like to talk about your needs regarding the care of your illness.

1. To what extent have you already talked to other people about problems with the treatment or care of your condition?

- Exchange in real life or via patient forums on the Internet
- Have you had conversations with family members or friends?

1. What (other) topics have you discussed?

- Lack of information from the doctor
- Lack of communication with doctor
- Difficulty finding an appointment
- Dissatisfaction with current therapy
- Influence of illness on the psyche
- Frequency of doctor's appointments
- No chance to ask questions

1. To what extent are you satisfied with the care you receive from your doctors?
2. What is rather positive in the care?
3. What is less satisfactory in the care you receive?
4. What is missing for a perfect doctor-patient conversation?
5. To what extent are you satisfied with the communication with your doctor?
6. To what extent are you satisfied with the processes in your care?
7. To what extent are there issues where you specifically need help but do not receive it or find it difficult to get it?

- Organization of the treatment plan
- Gaps in knowledge

1. **Potential benefits and preferences of digital applications**

Now we want to talk more specifically about digital health interventions. We have already collected some information from you in advance to see how much experience you have already gained with them:

*Almost all/some/some people* in the group have a smartphone or tablet and *all/most/some of you* use common applications, such as social networks, and all/most/some of you also use them for issues relating to your health. The group is therefore well mixed/almost uniform in this respect.

When we talk about digital health interventions, we mean apps, internet portals, online applications, or wearables (e.g., smartwatches) for your smartphone, tablet or computer that support you or your doctor in diagnosing, monitoring, or treating your dermatological disease or a concomitant disease.

- 1. Do you have a question about this definition?

I will now present several digital health applications for dermatology so that we can discuss them with you. Some applications exist, others we have adapted where necessary - they are therefore potential applications that we have simply put together as examples. We have used examples from the scientific literature as a guide.

**Application 1: Medication tracker**

Imagine a digital application that helps you to follow your treatment or medication plan. The application reminds you to apply an ointment or take tablets regularly by sending you a reminder message (e.g., three times a day). Taking the medication can then be confirmed by pressing a button in the application. Sometimes you may also be asked to send a photo of the application of the ointment, for example. It has already been shown that patients are more likely to adhere to the doctor's treatment plan.

a) To what extent can you imagine using such an application in your daily treatment routine?

i) What additional functions/standards would these applications need to have for you to use them regularly?

ii) What could possibly prevent you from using such an application?

- Time
- too complicated or time-consuming

**Application 2: Patient monitoring**

Imagine there was an application that recorded all the important data about your treatment and illness, allowing you to monitor the progress of your treatment. As a patient, you can enter a lot of important information. For example, you can regularly answer questions about your quality of life or satisfaction with your current treatment, keep a symptom diary, upload photos or content from other applications (e.g., from fitness trackers, pedometers, sleep trackers). You can then view the entries at any time, even over time, and you can discuss the data with your doctor or make it available to them.

a) To what extent can you imagine using such an application for your everyday treatment?

i) What additional functions/standards would this application need to have for you to use it regularly?

ii) What could possibly dissuade you from using such an application?

iii) To what extent can you imagine using such data in a doctor-patient discussion?

Imagine that your doctor could enter or upload additional information into this application, such as current medication or clinical data, e.g., the severity of the disease. The doctor's data could then be merged with the data you have entered, and you would have an even more detailed follow-up.

a) To what extent can you imagine using such an application for your everyday treatment?

i. To what extent can you imagine using such data in a doctor-patient discussion?

**Application 3: Digital communication with doctors**

Imagine a digital application that allows you to ask your doctor specific questions in writing about laboratory results, the form of therapy or symptoms that are occurring - similar to an e-mail or an instant messenger such as WhatsApp/Telegram/Threema. The doctor would reply within a certain time frame.

a) To what extent can you imagine using such an application for your everyday treatment?

i) What could possibly dissuade you from using such an application?

ii) What additional functions/standards would this application need to have for you to use it regularly?

iii) What (other) situation must be present for you to use a video conference?

b) To what extent would the storage of data or access to data by others be relevant to your use of the application?

The option of video consultations is becoming increasingly established. This allows you to attend your doctor's appointment via video conference. All you need for the meeting is an internet-enabled device.

a) To what extent can you imagine using such an application for your everyday treatment?

i) What could possibly dissuade you from using such an application?

ii) What additional functions/standards would this application need to have for you to use it regularly?

Now imagine a digital application that allows you to contact another dermatologist or a dermatologist you do not know, e.g., for a second opinion or for other dermatological complaints. You can upload pictures of your skin problem to an app or online application, fill out a short questionnaire on symptoms, the disease or current treatment and describe your complaints in writing. A dermatologist will then assess your case, ask questions if necessary and give you a specialist assessment within 48 or 72 hours - either a recommendation for treatment including a prescription or a referral. You will also receive a doctor's letter.

- 1. To what extent can you imagine using such an application?
     - Second opinion
     - Vacation
     - Something acute

i) What could possibly prevent you from using such an application?

ii) What additional functions/standards would this application need to have in order for you to use it regularly?

- 1. To what extent can you imagine using such an application if you have to pay for the consultation yourself?

**Application 4: Self-management and education**

Imagine a digital application that provides you with information about your condition and therapies, including specific tips and instructions on how to better manage your condition in everyday life, e.g., understandable information about the diagnosis and therapies or tips on how to deal with stress and psychological impairments caused by your condition. This can be done with the help of texts, videos, or pictures.

a) To what extent can you imagine using such an application?

i) What could possibly dissuade you from using such an application?

ii) What additional functions/standards would this application need to have in order for you to use it regularly?

b) Which of the topics mentioned would you find particularly interesting, or which additional topics would be of interest to you?

**Trade-OFF of all applications**

Unfortunately, it is not possible to develop all applications at the same time.

a) We would therefore like to know which of these applications you would prioritize. Could you explain why each application is particularly important to you? As a reminder: the following applications were presented and discussed:

- Medication tracker

- Patient monitoring

- Digital communication with doctors (video consultation, store-and-forward)

- Self-management and education

**Doctor-patient interaction**

After discussing many applications:

a) What do you think: To what extent can the use of digital applications change your relationship or communication with the dermatologist?

**3) Acceptance of digital health applications**

Now that you are familiar with our examples, but you may also be familiar with other digital health interventions:

a) How do you feel about digitalization in healthcare in general?

- - - - - Positively open-minded, as you can contact doctors more quickly, for example
        - Negative, as you are concerned about data protection with such applications

b) What proof of the quality and benefits of the application must be available for you to use it?

- Good rating in the App Store / Google Play Store
- Certification from TÜV (Technical Inspection), Stiftung Warentest (consumer organization providing impartial and objective information), Scientific evidence
- Recommendation from doctors

i) To what extent would you obtain information in advance? (Online, doctors, friends, other people affected)

ii) If your doctor recommends the application, would you use it without restriction?

c) How do you rate the issue of data protection when using digital applications?

d) Who should own which of the collected data and where should which data be stored?

- Patients
- Doctors
- Others
  1. To what extent would you make data available for research?

**4) Barriers and facilitating factors for the use of digital applications**

Think again about all the digital applications with which you are now familiar.

a) What could prevent you personally from using these digital applications?

i) In your opinion, what are the reasons for not using the digital applications?

ii) What are the disadvantages of digital applications?

b) What could encourage you personally to use these digital applications?

i) In your opinion, what are the reasons for using digital applications?

ii) What are the advantages of digital applications?

**5) Your own ideas and experiences**

a) Finally, as you are the expert on your condition, we would like to know if you have any other ideas for features or a digital application that can help you manage your condition?

i) Think about the topics you have discussed with other patients, friends, or family.

ii) Are there things that you still document by hand today that can be easily digitized?

- An illness diary

b) To what extent do you still use digital applications/functions that we have not yet discussed?

i) What was positive?

ii) What was negative?

c) To what extent can everyone else imagine using the described application for themselves

Do you have any comments or questions?

Thank you very much for your participation!
