# Supplementary material for: Exploring Acceptability, Barriers, and Facilitators for Digital Health in Dermatology: Qualitative Focus Groups With Dermatologists, Nurses, and Patients
Source: JMIR Dermatol. 2024 Sep 3;7:e57172. doi: 10.2196/57172 (PMC11408893; doi:10.2196/57172)
Supplement: Multimedia Appendix 3 [file derma_v7i1e57172_app3.docx]

**Overview of categories per stakeholder group and representative quote for the category**

**Table S1.** Themes, main, and subcategories—patient perspective.

| **Theme A: Attitudes Towards Technology** | **Main – Subcategories** | **Representative Quote** |
| --- | --- | --- |
|  | **Positive** | |
|  | Interest in utilizing digital health interventions | (G6; P4): ”All presented interventions are already interesting and I will, uh would have no inhibitions about actually trying those.” |
|  | Acceptance of digital health intervention if used complementary to in-person consultation | (G2; P2): “So I would find such an app good, but it must be complementary to the physicians’ consultation and not that a doctor would say, "Just take a look on the app," so to speak, in order to shorten parts of the consultation or the treatment time.” |
|  | **Negative** | |
|  | Not willing to switch dermatologists for lack of offering digital health interventions | (G3; P6): “I would also be reluctant to change my doctor, let's say. I wouldn't do that, because I'm satisfied. And I wouldn't do it just because of this app.” |
|  | Fear of data misuse by third parties | (G3; P4): “What happens if I lose my phone? Or someone hacks into it or something? That's all very private and it's not really anyone's business, so I don't really want to have it on my phone.” |
| **Theme B: Performance Expectancy** | **Positive** | |
|  | Greater involvement of patient in treatment | (G5; P2): “But if it goes the other way, as we just discussed, that we as patients then take a little more responsibility, and then you can present a condensed summary to the doctor, then maybe it will make sense.” |
|  | Improvement of patient-physician relationship | (G4; P3): “So, I think that it can improve the relationship even further. Also, trust can be strengthened. Trust in the dermatologist, but also trust in the therapy.” |
|  | Improvement of follow-up consultations | (G3; P3): “So the more data a doctor has about the patient, the better he can follow up.” |
|  | Reduction of unnecessary travel to medical appointments | (G4; P2): “I could avoid a trip, and I would not have to sit in the waiting room. I would consider that to be very positive.” |
|  | Usefulness of data for research purposes | (G6; P3): “So I find the whole topic of digitization so interesting from the data aspect, in order to generate results from it with data analytics, etc., from which both the doctors and the patients can benefit, um, yes, I'll say that. Um, that's what I find so charming about the whole thing when you digitize it.” |
|  | **Negative** | |
|  | Impersonal patient-physician relationship | (G6; P4): “The personal contact could suffer a bit... that my doctor... I've known him for ages... So we talk, if there's time, about the children or just things on the sidelines. Things that don't just have to do with the disease. That would naturally fall by the wayside a bit.” |
|  | **Requirement** | |
|  | Additional value for both patient and physician | (G1; P3): “I think we have to look at all these apps in general and see what the advantage is. Where is the advantage for me as a patient? But maybe also where is the advantage for the doctors. What advantage does the doctor have when I use such a digital app? Does he have any advantages at all?” |
| **Theme C: Effort Expectancy** | **Negative** | |
|  | Low digital competencies among older patients | (G5; P1): “That could be practical [...], but if they [older patients] can't operate a smartphone at all, it won't help.” |
| **Theme D: Social Influence** | **Positive** | |
|  | Trust in physician’s’ recommendations | (G5; P2): “It doesn't need a seal of quality, I would trust an App, which my clinic or physician suggests to me. Then I would be pretty sure that it is good.” |
|  | Trust in recommendations digital health developments of/by trustworthy institutions | (G5; P5): “I would need to see: Okay, there are institutes behind it, there are associations behind it and health insurance companies accept it. Things like that. And it should be certified.” |
|  | **Negative** | |
|  | Dependence on physicians’ acceptance | (G2; P2):“The practitioner […] plays the main role. Because with him is the main interaction and he is the main person to whom one would turn, be it digital or otherwise. So if the- if the doctor rejects digitization, then there's no point to any of this. So then it's a side event.” |
| **Theme E: Facilitating Conditions** | **Facilitator** | |
|  | A single application for different purposes | (G1; P3): “The described applications are all a piecemeal. I can make a constellation here, I can get a second opinion here, I can at best download my prescription to my cell phone instead of in paper form. Um, I have to lug doctor's letters from A to B, then there's faxing, so I would prefer an integrated solution, whether that's for dermatology or for anything else.” |
|  | Clear data access permissions | (G1; P2): “I think it's important to clarify, who has access to this data. I think that is the most important thing. Who has access to it? […] I think that is always a critical point, because especially in patient data there can be a lot of information in it, which- where a lot of others would be interested in it.” |
|  | Possibility to choose between analog and digital health intervention | (G1; P1): “I always think that this option would be nice. So that you have the option of using something digital, but at the same time you also have the option of continuing to use analog.” |
|  | Sufficient reimbursement | (G6; P4): “There is only the question of costs in all the things you mentioned, that should be clarified. […]. As I said, if it’s technically possible and financially possible, I'm in.” |
|  | **Barrier** | |
|  | Utilization of outdated technology in practices | (G2; P1): “But what strikes me are some common things. For example, doctors still use faxes an awful lot here or in derma. So, for me it is an antiquated device, and my son doesn't even know what a fax is, yes. But because he has never seen it before.” |
|  | Current data security regulations impede the functionality of digital applications | (G5; P2): “What makes me only sometimes a bit skeptical, also my experience from Denmark, where all is digitalized. I think it's great when there aren't so many difficulties, like here, where you always have to ask so much permission everywhere and all the offices don't work together. That makes the whole thing cumbersome.” |

**Table S2.** Themes, main, and subcategories—physician perspective.

| **Theme A: Attitudes Towards Technology** | **Main – Subcategories** | **Representative Quote** |
| --- | --- | --- |
|  | **Positive** | |
|  | Dermatologists are required to participate in digitalization to have a voice in shaping the system | (G2; D2): “That's - we have to participate, of course; we can opt out, yes, and say: We don't want that, because - The caravan moves on, doesn't it, and with us or without us, doesn't it, and so it's better that we're there and can at least, meaningfully help shape that, yes.” |
|  | Higher acceptance among younger patients | (G3; D1): “The patients who enter that [time to take medication] into the smartphone as a reminder; that's what the younger people do.” |
|  | Acceptance of digital health intervention if used complementary to in-person consultation | (G3; D6): “I think this would never, never really replace a consultation; it can only be a supplement.” |
|  | **Negative** | |
|  | Fast pace of digitalization makes life more difficult | (G2; D2): “And then at some point the computer came along, and people thought, yes, with computers we have a simplification, then I certainly have more time for the patient. Exactly the opposite has happened: I have more and more possibilities, more and more apps, but I'm overwhelmed by emails, apps, all kinds of things, and time is getting faster and faster, and the systems are getting faster. I can't keep up at all, I'm more and more confused. And all you hear about everywhere is burnout and whatnot. So, what makes life easier with digitalization - in the end, it makes life more difficult. “ |
|  | Fear of being replaced by digital health intervention | (G6; D4): “On the one hand, you want to be tech-savvy in the digital field, you want to keep up with the times. On the other hand, even as a doctor, you have the worry that this digitalization of machines will eventually replace us. For example, we know that rheumatologists and radiologists will soon no longer be able to work properly because artificial intelligence can make much better assessments than radiologists.” |
|  | Fear of data misuse by third parties | (G2; D1): “And if somebody - Yes, and I would -, I -, I assume that ultimately all the data that is collected will be made available to insurance companies. That our risk assessments will be changed, that our costs will be changed. It's not going to be to everybody's advantage, it's going to be against us as well. There's nothing you can do about that, but whether we should be pushing that unbiddenly to get it there quickly, I don't know.” |
|  | **Neutral** | |
|  | Patients are unrestrained towards their data privacy | (G5; D1): “So patients are self-indulgent when it comes to privacy. Of their own accord. They send you naked photos via WhatsApp. When you get a cell phone, everyone thinks I'm a child pornographer, because there are so many baby bums from vacation with diaper dermatitis on it. So they are completely, completely uninhibited.” |
|  | Physicians rate personal impressions over evidence | (G4; D4): “Yes, well, I don't know if I need the evidence. So I would probably have to look at the app and would first decide according to my feeling yes. So I (have to say)- what seems logical to me.” |
|  | The dermatologist sees no need for adjustments | (G1; D5): “Because we're not patient-oriented, we're disease-oriented and we don't need to offer another service, we don't need that at all. We are the rare good and to the rare good and we need meaningful solutions and not more nonsense.” |
| **Theme B: Performance Expectancy** | **Positive** | |
|  | Greater involvement of patient in treatment | (G1; D3): “So with many of these digital applications you have to say, they are motivationally quite useful. Like a health app, were I have to make my daily steps” |
|  | Improvement of patient-physician relationship | (G5; D1): “It can only improve [the relationship]. Because we have more time for important patients and unnecessary consultations can be reduced. I think that would be positive” |
|  | Promotion of need-based care | (G3; D4): “Because that would give us more time for more important patients and we would be relieved for the less important issues. I think that would be positive.” |
|  | Promotion of standardized care | (G5; D2): “Yes, and that's why I say that it would also help to make the field of expertise more homogeneous again if we had tools like this, where practically everyone has the same advice and the same standards.” |
|  | **Negative** | |
|  | Impersonal patient-physician relationship | (G3; D4): “I think we must be careful that the relationship will not be worsened, while everyone will just look on their screen and not in each other’s faces” |
|  | Additional workload | (G3; D6): “So I think, that will not be a relief, that will be more work, so for example the people that we see by video consultation, yes most of them come anyway […]. So it costs more time and maybe you could do in the evening, when you really need to relax. I already have a (exhausting) day anyway, and then a video consultation in the evening?” |
|  | Overload of information | (G2; D2): “Yes, well, this flooding, that is a central problem, yes, and that in the end, in principle, often the important is no longer read or no longer reaches the addressee or the addressee just pushes it away. […] What used to be too little is now too much.” |
|  | **Requirement** | |
|  | Technical functionality should result in an added value | (G1; D4): “I want to have the computer as a help and not as a toy, as it is for a programmer, who tries to do tricks and things that we don't need at all.” |
|  | Additional value for both patient and physician | (G5; D4): “I don't need evidence. I just need an apparent added value for the patient and for me. And then I would recommend it.” |
| **Theme C: Effort Expectancy** | **Positive** | |
|  | High digital competencies among nurses | (G4; D3):“So these nurses, they love WhatsApp. So every nurse, even on night duty, has her cell phone in her bag.” |
|  | **Negative** | |
|  | Low digital competencies among older patients | (G6; D2): “In my opinion, 20-30 percent of humanity is still digitally illiterate. That includes people like my mother, who somehow managed to crash the Internet I think three times by now. Not the computer, the Internet.” |
|  | Difficulties to assess the integrity of applications | (G2; D2): “I can no longer tell whether it's serious or not, and what's the catch, the -. And it's all become so fast-moving. I don't have the time to spend two hours reading up on it. It has all become very difficult.” |
|  | Low digital competencies among nurses | (G4; D2): “For the cell phone at home, it's still enough for everyone. But when it comes to the practice computers, so at the latest from uh I would say 35 plus you no longer see yourself in a position to do so [work with a computer]. That is too complicated.” |
|  | Exclusion of digital illiterate patient groups from care | (G5; D1): “There are still people who don't have Internet access. You really have to be careful that you don't leave them behind.” |
|  | **Requirements** | |
|  | Easy to use applications | (G2; D3): “[…] and such apps have to be kept as simple as possible […]” |
| **Theme D: Social Influence** | **Positive** | |
|  | Trust in recommendations of colleagues | (G1; D2): “But I would be influenced by colleagues who tell me that this works well and that this is feasible.“ |
|  | Physicians rate colleagues’ recommendations over evidence | (G2; D5): “The most important evidence is still colleagues whom you trust and who also have experience, and there you listen to three, four opinions, and if that goes in one direction, then you try that.” |
|  | Trust in recommendations of physician associations | (G2; D2): “The online services will continue to be pushed and scientifically processed, at some point we will have other tools, and then I would also trust the professional association for the time being.” |
|  | **Negative** | |
|  | Dependence on patients’ acceptance | (G5; D2): “It is also up to the patient to use the app, yes. So, if a medical tracker exists where data of the patient is recorded and he does not want that, he does not have to do that.” |
| **Theme E: Facilitating Conditions** | **Facilitators** | |
|  | A single application for the same purpose used by all physicians | (G4; D5): “...we have to agree on which of the many systems we want to use uniformly. Otherwise, I'll have to use number one of these messenger systems for colleague A and number two for colleague B. Uh, and I think that's the main problem.” |
|  | General trust in data protection and security | (G5; D2): “If data security is secured, we have to have a bit of trust at some point, because otherwise it won't work. Otherwise, it won't work, yes.” |
|  | Sufficient reimbursement | (G4; D3): “We simply can't do that with this abundance. And that's why the legislators and the health insurance companies have to realize that if they want to do it, and we're back to the first point, then they have to put extra money in their hands to make it practically palatable for the doctor.” |
|  | **Barriers** | |
|  | Difficulty to integrate digital health interventions into busy daily routines | (G5; D2): “Well, I haven't done it either. I won't do it either. But that's usually because of the time factor. If you have so many functions in addition to your work in the practice, then you think: Do I still sit down there in the evening and answer something like that?” |
|  | High-maintenance IT-infrastructure | (G1; D2): “But we all have enough to do with our practical program and are somehow all just busy keeping it running and somehow installing the very latest that politics has come up with.” |
|  | Current data protection regulations impede the functionality of DHIs | (G1; D1): “This discussion about data protection doesn't bring us much at all, and unfortunately it doesn't have any advantages over the world at the moment, only disadvantages.” |
|  | Insecurity towards data privacy laws | (G2; D2): “But I'm so caught up in this topic. I say it all only because I have the feeling that it's getting more and more complicated, soon you're not allowed to say anything anymore, and if you've done something wrong, someone could report you or whatever. That's just no longer addressed the human site.” |

**Table S3.** Themes, main, and subcategories—nurse perspective.

| **Theme A: Attitudes Towards Technology** | **Main – Subcategories** | **Representative Quote** |
| --- | --- | --- |
|  | **Positive** |  |
|  | Digitalization in the medical field is deemed necessary | (G5; N3): “I think that something like this [digitalization] will be necessary, the capacities are simply no longer there.” |
|  | Acceptance of digital health intervention if used complementary to in-person consultation | (G6; N6): “I would find it super as a supplement. But only as a supplement.” |
|  | **Negative** | |
|  | Older patients prefer personal consultation | (G3, N2): “I think the older generation places ... uh... even more value on it, on the personal doctor-patient contact. You could feel... I think you could feel offended if you said, - No, we're going to do this online, we're not going to do it here in the practice anymore.” |
|  | Nurses prefer personal consultation | (G2; N3): “The whole counseling and so on personally I find as I said- I had already mentioned that I do a lot of counseling and through the counseling I do, I personally would miss- very, very much.” |
|  | Fear of data misuse by third parties | (G6; N1): “I have a few studies where it's just similar and that's exactly the problem that the patients point out to me, that, I don't want that, I also don't want that ...uh.... someone externally has access to my data.” |
|  | **Neutral** | |
|  |  |  |
|  | Economic concerns are important for decision making | (G4; N2): “So I actually looked first, the three portals, then I chose one where I saw Ok, that could be just also economically interesting thing for our practice, that plays nevertheless always a role” |
| **Theme B: Performance Expectancy** | **Positive** | |
|  | Greater involvement of patient in treatment | (G6; N1): “Perhaps one argument would be to give a bit of responsibility back to the patient and remind him that he has to work on his healing. I also see that as an advantage, i.e. when a patient has to actively enter something or has to deal with it. I think that's actually also an advantage, yes.” |
|  | Support of treatment process through digitized patient data | (G6; N5): “Data that you can get from the patient is helpful to adapt the following therapy.” |
|  | Time savings during the treatment process | (G5; N5): “… when it's digital, the doctor can immediately write in the medical history, I can prepare the prescription, the doctor is sitting in the treatment room, I'm sitting at the reception. He writes it in the medical history and I can prepare it at the same time, so ... For the patient, too, it's much, much, much shorter in terms of time.” |
|  | **Negative** | |
|  | Impersonal patient-physician relationship | (G4; N4): “On the other hand patients may lose the personal contact to a dermatologist or any other physician.” |
|  | Additional workload | (G6; N7): “So I think that it makes a lot of pressure, time pressure too. Then everything is always demanded so quickly, if you write an e-mail at 1 p.m., then you expect an answer at 2 p.m. and preferably for an appointment on the next day.” |
|  | **Requirement** |  |
|  | Additional value for both patient and physician | (G6; N1): “But it can also go in exactly the other direction, so that both sides really get added value out of it.” |
| **Theme C: Effort Expectancy** | **Positive** | |
|  | High digital competencies among younger patients | (G3; N1): “Totally good, especially for younger patients who use their smartphones or iPads every day anyway and always have it in their hand, always |
|  | Decreasing proportion of patients with low digital competencies over time | (G6, N6): “But the generation, the over-80s, yes, they are probably not so much tied to the smartphone. But I also have many patients who are much fitter than I would have suspected. I think that in ten years, that will be much less of these patients [not yet tied to a smartphone].” |
|  | **Negative** | |
|  | Low digital competencies among older patients | (G2; N2): “… for the older generation that will probably be difficult. But um from up to a certain age I think that is quite well received.” |
|  | Initial high effort to implement digital health interventions | (G6; N6): “As with everything that is new at first, it is of course a lot of work, a huge amount of work for those involved, but then I think there is also a great benefit underneath. Earlier, I think a colleague said in the context of documentation or digitization that at the beginning, of course, it was new and incredibly difficult, and everyone said, oh God, oh God, oh God, and now everyone says, thank God we have it much nicer now.” |
|  | Low digital competencies among older physicians | (G6; N5): “But it was already foreseeable in the time of digitization how it will become, so it was quite good so far and also basically, we also have many or a few older physicians with us who also had strong problems with it.” |
|  | **Requirement** | |
|  | Easy to use applications | (G3; N1): “It shouldn't be so complicated. […] but if that ... uh... somehow an App is, I download, there I must enter then once a password and then, bang I can set my question, I would say, yes, that is yes simple.” |
|  | Easy integration into daily routines | (G5; N3): “Application that is doable, that does not take so much time, I say that you can integrate well into the workday, … […] That would be an application I could imagine to use.” |
| **Theme D: Social Influence** | **Positive social influence** | |
|  | Trust in physician’s recommendations | Interviewer: “If it is really recommended by the doctor – do you think more patients would use a DHI?”  (G3, N2): “Uh..., yes, I think so, yes,” |
|  | Trust in the recommendations of colleagues | (G5; N5): “and I looked at it which dermatologists are still represented, and then I just saw some of their old circles there, then I thought yes ok, maybe you could ask on their experience.” |
|  | **Negative** | |
|  | Dependence on physicians’ acceptance | (G4, N3): “Basically, it's the doctors who decide, and depending on the century the doctors come from, I'll say quite profanely, you can say yes, we as a practice go along with it. The nurses can be as old or as young as they like. The doctors basically have to say 'Yes, I can imagine' or 'No, they've always done it that way and we'll continue to do it the way it's going right now.” |
|  | No trust in nurses’ recommendations by patients | (G3; N2): “It doesn't matter if it's a recommendation for something, if it's just a cream for something, if it's something I say at the front, it's only something she says. But ...uh... if the doctor says it in the treatment room, then, then it's great and then it's like that and then we'll definitely buy it.” |
| **Theme E: Facilitating Conditions** | **Facilitator** | |
|  | A single application for different purposes | (G3; N3): “Everything has to be compatible, otherwise it won't work.” |
|  | Engagement of nurses in digital processes | (G3; N3): “I would have liked to offer video consultation hours. You can also really do it in such a way that a doctor is present, an MFA is present, and that all the other things, i.e. writing and doing, i.e. writing materials, etc., are taken over by the MFA, that's not a problem. And so that the communication of doctor and patient takes place, that could have worked. But it shouldn't be. So yes, too bad.” |
|  | Patients possess digital devices | (G5; N4): “That would actually be utilized because people certainly now everybody has, ultimately a cell phone.” |
|  | Sufficient reimbursement | (G6; N7): “As I said, then cannot get reimbursed, because the system does not allow it.” |
|  | Pandemic has accelerated the progress of digitalization | (G5; N2): “Well, Corona has now shown that many things can be done digitally. So that's where it sits. I think it will go more digital and then you really have to think about how you can integrate certain things.” |
|  | **Barrier** | |
|  | Difficulty to integrate digital health interventions into busy daily routines | (G6; N7): “Not for our practice, the doctors don't take the time, the, if then they want to see that you can bill for it and to more, we are not at the moment. We are so so busy or overloaded -.” |
|  | **Neutral** | |
|  | Data privacy is very important in medical practices | (G6; N4): “So for us the most important aspect is the data security of our patients.” |
